# NBA team home advantage: Identifying key factors using an artificial neural network

**DOI:** 10.1371/journal.pone.0220630

**Published:** 2019-07-31

**Authors:** Austin R. Harris, Paul J. Roebber

**Affiliations:** Atmospheric Science Program, Department of Mathematical Science, University of Wisconsin–Milwaukee, Milwaukee, Wisconsin, United States of America; Instituto Politecnico de Viana do Castelo, PORTUGAL

## Abstract

What determines a team’s home advantage, and why does it change with time? Is it something about the rowdiness of the hometown crowd? Is it something about the location of the team? Or is it something about the team itself, the quality of the team or the styles it may or may not play? To answer these questions, season performance statistics were downloaded for all NBA teams across 32 seasons (83–84 to 17–18). Data were also obtained for other potential influences identified in the literature including: stadium attendance, altitude, and team market size. Using an artificial neural network, a team’s home advantage was diagnosed using team performance statistics only. Attendance, altitude, and market size were unsuccessful at improving this diagnosis. The style of play is a key factor in the home advantage. Teams that make more two point and free-throw shots see larger advantages at home. Given the rise in three-point shooting in recent years, this finding partially explains the gradual decline in home advantage observed across the league over time.

## Introduction

Home advantage is unanimously accepted as a key factor to a team’s success in a game. It’s openly discussed by coaches, players, and fans. Academics have observed it in nearly all team sports. Some attribute this phenomenon to crowd noise [1–4], where rowdier crowds increase the advantage for the home team, possibly influencing referees in the process [1, 3]. Others attribute it to fatigue from away travels [5], familiarity with one’s own facilities [6], rest between games [7], referee bias [1, 8–9], and altitude [10]. Despite these studies, no definitive explanation currently exists.

National Basketball Association home advantage is particularly interesting. All games are played indoors, removing any influence from the weather. Unlike baseball, there are no differences between home courts. Still the NBA home advantage is the highest of all sports, with European soccer as the only exception [11]. We know the NBA home advantage mostly comes within the first quarter of games or when the home team trails [10, 12] and that it has declined across the league with time [6, 10, 12–13]. But *why*? One theory attributes the decline to reduced referee bias from increased use of video-replays [10]. Another attributes the decline to reduced crowd-support from a homogenization of the audience [13]. We also know the style of play has changed significantly over time, with three-point shots becoming increasingly popular. Could this be associated with changes in the home advantage with time?

The home advantage varies by team [14]. The Denver Nuggets and Utah Jazz have the highest home advantage [10], a finding attributed to the high altitude of these cities where home teams are more acclimated to its effects on the body. Meanwhile larger market organizations, like the New York Knicks, might have a slight advantage over their smaller market counterparts, like the Indiana Pacers [15]. These factors may contribute to the advantage; however, they are static and likely cannot explain league-wide changes in the advantage with time.

In this study, we investigate whether team season performance statistics, such as total points scored, two and three-point shot attempts, field goal percentage etc., contribute to the home advantage and whether they explain the decline in the advantage with time. In addition, we examine the extent to which known contributors (NBA market size; crowd noise; physical elevation) influence the advantage. The chosen approach is to diagnose home advantage using an artificial neural network. Through this we will show that a team’s home advantage is largely accounted for by the types of shots that it makes, reflecting a particular style of play, and that this relationship partially explains the decline in the advantage with time.

## Methods

### Available data

NBA Team performance statistics were obtained for 32 regular seasons spanning most of the 3-point era (1983–84 to 2017–18). Season statistics were chosen to easily examine league-wide changes over time. Preseason and playoff games were not included, nor were the 1998–99 and 2011–12 seasons which were shortened by lockouts. These data were downloaded directly from https://www.basketball-reference.com/play-index/tgl_finder.cgi (select: search for cumulative season games matching criteria). Specific statistics available are: number of wins, number of losses, field goals made, field goal attempts, field goal percentage, two-point shots made, two-point shot attempts, two-point shot percentage, three-point shots made, three-point shot attempts, three-point shot percentage, free-throw shots made, free-throw shot attempts, free-throw shot percentage, and total points scored. Downloading data for both home and away games provides shooting statistics in four categories: season performance at home (hereafter Home), performance away (hereafter Away), opponent performance at home (hereafter Home Opp), and performance away (hereafter Away Opp).

In addition to the team performance statistics, season attendance records were obtained for 18 seasons (2000–01 to 2017–18) from ESPN. These data were downloaded directly from http://www.espn.com/nba/attendance. Specific statistics available were: home team attendance, percent of stadium filled at home, away team attendance, and percent of stadiums filled away. To serve as a potential proxy for market size, metropolitan population data were downloaded for NBA cities for each decade (https://census.gov). NBA city elevations were found via *USGS* (https://pubs.usgs.gov/gip/Elevations-Distances/elvadist.html).

### The approach

There are multiple ways to define home advantage. Point differentials [10] are an effective approach for quarter and play-by-play stats. For season statistics, win shares [16] are one approach. The most common definition is to divide the number of home games won by the total games won in a season [10, 12]. One shortcoming to this definition is that the advantage is particularly sensitive to changes when the denominator is small. This produces a disproportionate amount of noise in the data for teams with less wins overall. To avoid this issue, we propose a similar definition involving the difference in win percentage at home and away:

$$Home\;Advantage=\left(\frac{Home\;Wins}{\;Total\;Games\;Played}\right)-\;\left(\frac{Away\;Wins}{\;Total\;Games\;Played}\right)$$ (1)

The distribution of the observed home advantage is shown in S1 Fig.

An artificial neural network was developed to diagnose the advantage using the software, JMP Pro. Though similar to multiple linear regression, neural networks are preferred when non-linearities in the data may be important and we do not wish to specify their structure (e.g., using products between inputs). This was the case with our dataset, as shown in the next section. The first step in building the network was to split the data into training and cross validation datasets to prevent overfitting the data. Since the home advantage changes with time, balancing the data was necessary and achieved by randomly assigning roles from nine pre-defined eras using a Monte Carlo simulation (S1 Table). The second step was to identify the most useful input variables. This was done by sequentially removing the worst performing predictors after each model was built until maximum diagnostic performance was achieved (highest R^2^) in the cross-validation data. Once this final model was established, a sensitivity analysis was performed on the model to understand how the inputs combined to diagnose the advantage. Specifically, we measured the change in the home advantage when each input is increased and decreased by ten percent. In the following section, we will show: 1) the model’s ability to determine the advantage 2) which inputs were the most important variables and 3) how the elements combine to diagnose the home advantage.

## Results and discussion

### The ideal model

**Fig 1:**
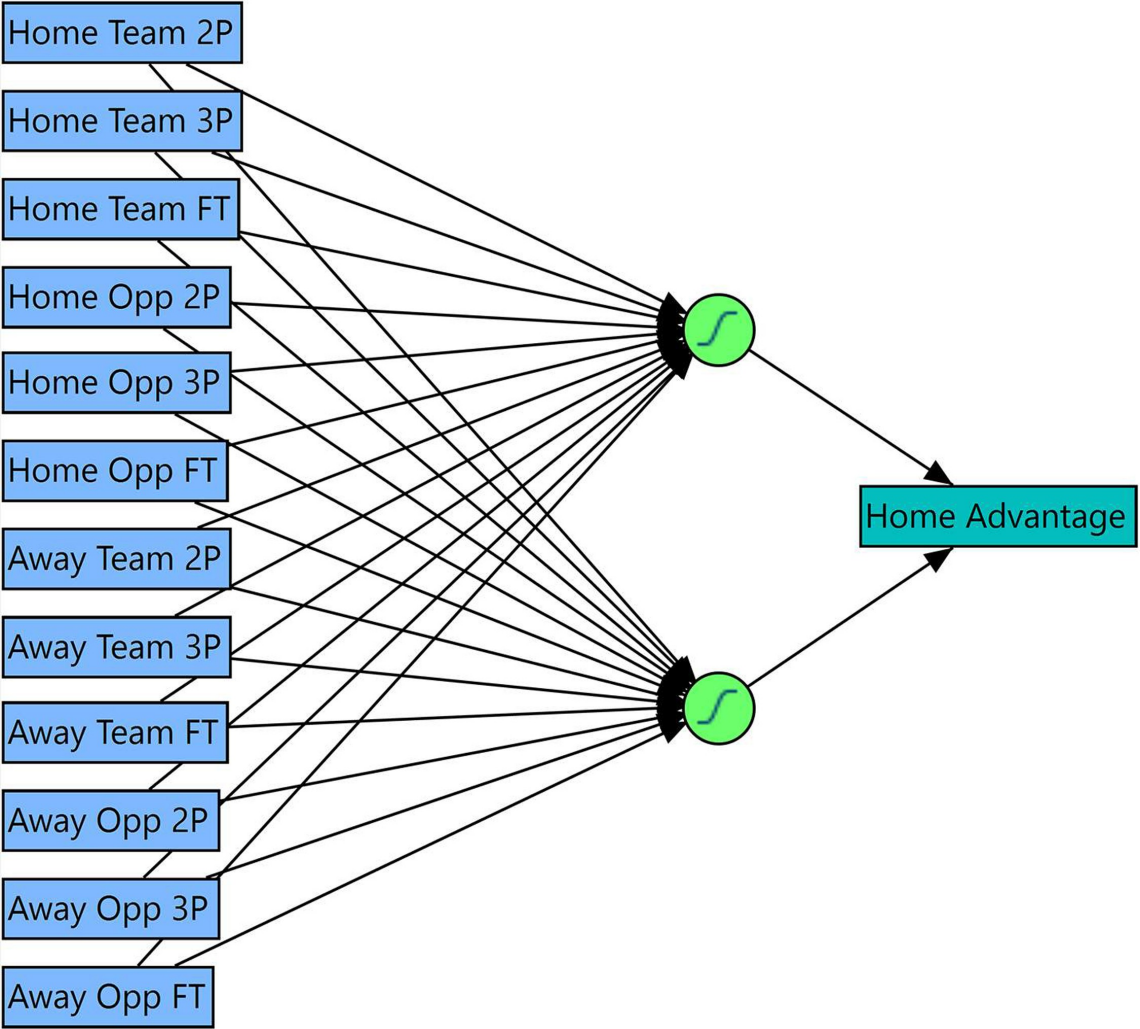
ANN schematic. A schematic showing the 12 best performing inputs (blue), the ideal number of nodes (green), and the diagnosed home advantage (teal).

The performance of neural networks with various inputs is shown in Table 1. The best performing neural network is a two-node, single hidden layer perceptron (MLP) network with the following twelve inputs: Two-point (2P), three-point (3P), and free-throw (FT) shots made in a season by Home, Home Opp, Away, and Away Opp (Fig 1). All twelve inputs are essential to the model’s success: removing any one input reduces the model performance significantly. This ideal model accounts for most of the variance in the data and generalizes well between the training and cross-validation datasets (R^2^ = 0.7 for both). The Mean Absolute Error (MAE) in the model is 0.051, while the 25^th^ and 75^th^ percentile MAE’s are 0.020 and 0.073, respectively, indicating that the model performs well.

**Table 1:** Model performance.

Model Performance (R^2^)	Single-layer MLP (12 inputs)	Single-layer MLP (all inputs)	Two-layer MLP (12 inputs)
Training	0.7	0.66	0.71
Cross Validation	0.7	0.63	0.71

Training and cross-validation model performance (R^2^) is shown for: 1) The ideal, single-layer MLP neural network used in the sensitivity analysis 2) An identical, single-layer MLP neural network that includes all available inputs selected for the study 3) A two-layer MLP neural network with the preferred 12 inputs.

Networks that include shot attempts, shooting percentage, total points scored, field goals, attendance statistics, elevation, and market size as predictors added no improvements in performance. Adding a second layer to the network adds modest, but insignificant improvements (Table 1). Although not shown in Table 1, adjusting the number of nodes and activation functions decrease network performance slightly for all models (0.6 < R^2^ < 0.7 for both). Multiple linear regression models perform worse than the neural networks, regardless of the inputs (R^2^ < 0.5), verifying the need for a neural network approach (not shown).

**Fig 2:**
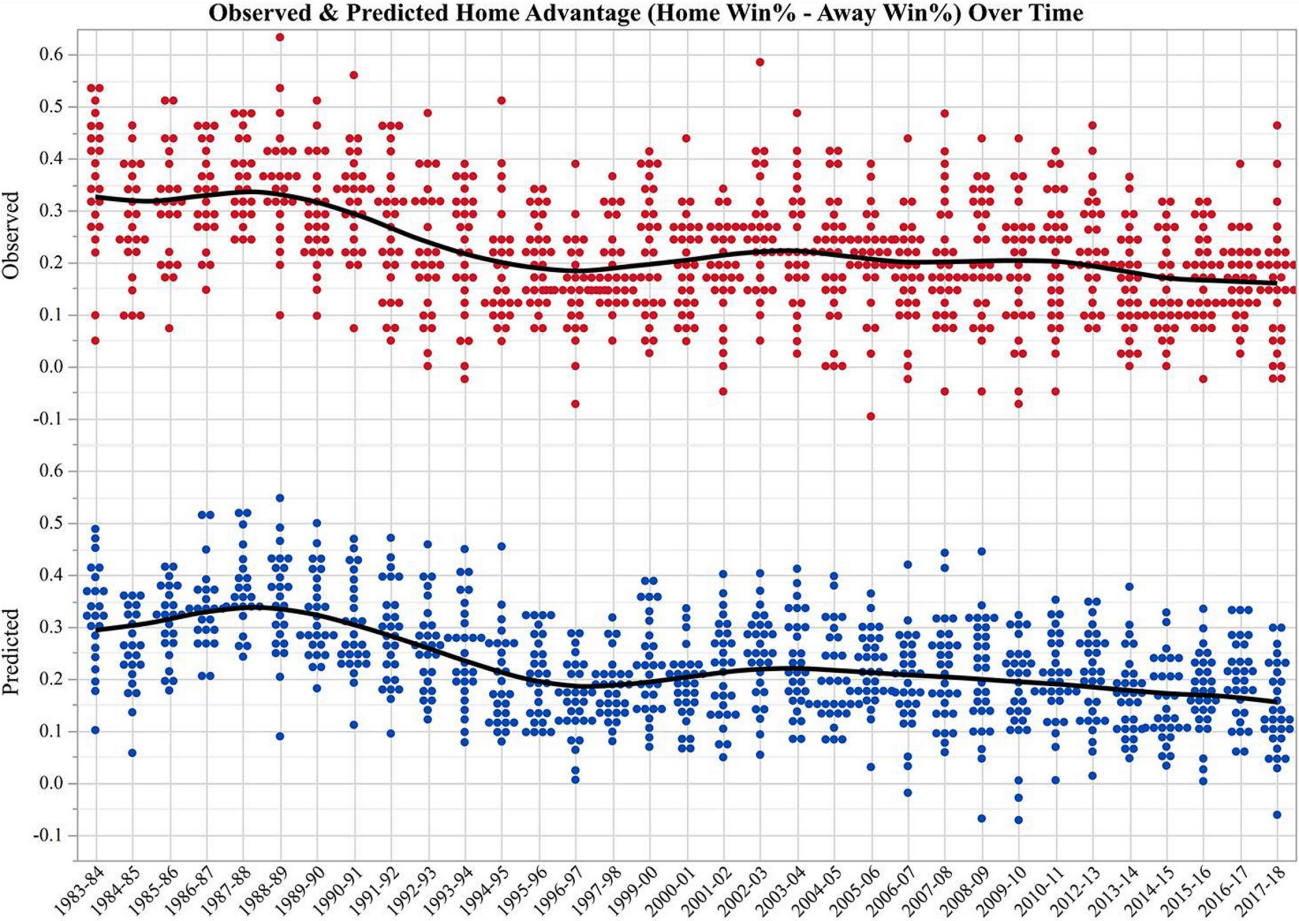
Predicted and observed home advantage over time. Observed (red) and diagnosed (blue) home advantage with a best fit line (black).

**Fig 3:**
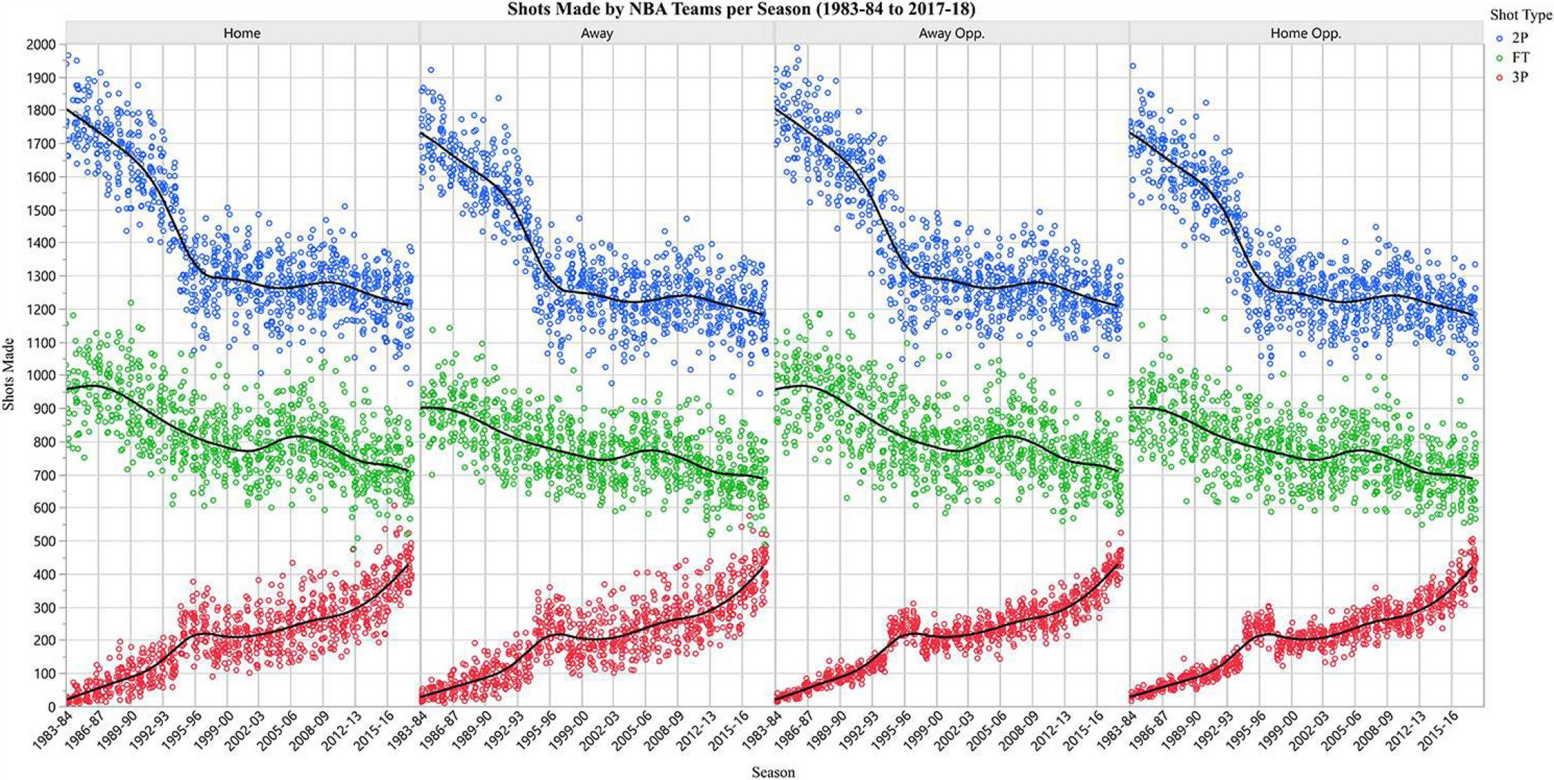
Twelve ANN inputs over time. 2-point (blue), free throws (green), and 3-point (red) shots made by the team at home (Home), their opponent at home (Home Opp), the team away (Away), and their opponent away (Away Opp).

The observed home advantage decreases over time in our dataset, a finding consistent with previous studies and home advantage definitions [6, 10, 12–13]. Specifically, the advantage peaks in the late 1980s, has a relative minimum in the mid-1990s, with perhaps a slight increase in the early 2000s, and has steadily declined since then (Fig 2). The diagnosed home advantage successfully captures these changes (Fig 2). Fig 3 shows how the model inputs evolve with time. 2P and FT makes decline throughout the dataset while 3P steadily increase. These changes are especially drastic in the mid-1990s when the observed advantage reaches a minimum. These indicate fundamental changes in how the game is being played, and these changes are linked to the changes in the home advantage with time.

### Sensitivity analysis

The goal of the sensitivity analysis is to see how the inputs combine, thereby providing insight into the origins of the home advantage. The analysis was performed for when the advantage is high (75^th^ percentile), low (25^th^ percentile), and average (50^th^ percentile). Due to the evolution in the inputs with time (Fig 3), the analysis was performed separately for early and late eras. Results are presented in Table 2.

**Table 2:** ANN sensitivity analysis.

**Early Era** **Cases**		High HA (75^th^ Percentile)		Average HA (50^th^ Percentile)		Low HA (25^th^ Percentile)	
		+10%	-10%	+10%	-10%	+10%	-10%
Home	2P	**0.223**	**-0.283**	**0.155**	**-0.179**	**0.251**	**-0.304**
	3P	0.004	-0.004	0.010	-0.010	0.005	-0.005
	FT	0.065	-0.080	0.010	-0.024	0.059	-0.072
Home Opp	2P	**-0.257**	**0.207**	**-0.153**	**0.136**	**-0.272**	**0.230**
	3P	-0.011	0.011	-0.006	0.006	-0.010	0.010
	FT	-0.081	-0.065	-0.049	0.047	-0.075	0.072
Away	2P	**-0.278**	**0.168**	**-0.250**	**0.302**	**-0.282**	**0.238**
	3P	-0.006	0.006	-0.021	0.021	-0.007	0.007
	FT	-0.063	0.060	-0.066	0.070	-0.073	0.071
Away Opp	2P	**0.147**	**-0.278**	**0.297**	**-0.250**	**0.215**	**-0.272**
	3P	0.007	-0.007	0.013	-0.012	0.006	-0.006
	FT	0.082	-0.091	0.090	-0.085	0.085	-0.088
**Late Era** **Cases**		High HA (75^th^ Percentile)		Average HA (50^th^ Percentile)		Low HA (25^th^ Percentile)	
		+10%	-10%	+10%	-10%	+10%	-10%
Home	2P	**0.186**	**-0.231**	**0.172**	**-0.191**	**0.185**	**-0.201**
	3P	0.031	-0.032	0.056	-0.057	0.097	-0.010
	FT	0.043	-0.054	0.058	-0.065	0.068	-0.076
Home Opp	2P	**-0.175**	**0.148**	**-0.181**	**0.165**	**-0.196**	**0.182**
	3P	-0.050	0.047	-0.05	0.045	-0.093	0.089
	FT	-0.057	0.058	-0.066	0.064	-0.060	0.056
Away	2P	**-0.230**	**0.176**	**-0.186**	**0.150**	**-0.178**	**0.166**
	3P	-0.041	0.038	-0.057	0.052	-0.101	0.095
	FT	-0.057	0.055	-0.055	0.053	-0.066	0.065
Away Opp	2P	**0.149**	**-0.200**	**0.146**	**-0.200**	**0.164**	**-0.181**
	3P	0.061	-0.067	0.040	-0.042	0.091	-0.096
	FT	0.067	-0.070	0.066	-0.070	0.052	-0.053

Shown values are the change in the predicted home advantage when the inputs are changed by +- 10%. The statistic (out of 2P/3P/FT) with the highest percent change is shown in bold.

The key findings from this analysis are:

- *2P*, *3P*, *and FT*: 2P is the strongest shot predictor of the home advantage. FT made are more important than 3P in the early era, while equally important in the later era.
- *Home and Home Opp*: The diagnosed home advantage is increased when 2P, 3P, and FT are increased for Home and decreased for the Home Opp. This implies that the *better* a team performs at home–both on the offensive AND defensive end–the larger the advantage.
- *Away and Away Opp*: The diagnosed home advantage is increased when 2P, 3P, and FT are decreased for Away and increased for the Away Opp. This implies that the *worse* a team performs away–both on the offensive AND defensive end–the larger the home advantage.

## Discussion and conclusion

What determines a team’s home advantage? Is it something about the team, the crowd, or the home arena? And why does it change with time? We have found that a team’s advantage can be determined using an artificial neural network with 2P, 3P, and FT shots made by Home, Home Opp, Away, and Away Opp as inputs. Contrary to previous work, attendance [1–4, 13], elevation [10], and market size [15] were not relevant to understanding home advantage, nor were shot attempts, shooting percentage, overall W-L%, and total points scored. Observed changes in the 2P, 3P, and FT made (i.e., transitions in the style of play) are responsible for the change in the advantage with time (Fig 3). This is the first known study to attribute shot type to the home advantage.

A sensitivity analysis on the neural network suggests teams can maximize their advantage–and hence their odds of winning–by employing different shot selection strategies when home versus away. When playing at home, teams can maximize their advantage by shooting more 2P and forcing opponents to take more 2P shots. When playing away, teams can minimize an opponent’s home advantage by shooting more 3P and forcing opponents to take more 3P shots. The adjustments in shot selection can be accomplished by changing the play-calling, defensive scheming, and the team personnel as seen fit. However, the adjustments should be considered within the greater context of shot selection strategies such as the efficiency of 3P versus 2P shots overall, skillsets of available players, team identity, and game-specific matchup advantages.

The study does not address *why* certain types of shots matter more to the home advantage–the granularity of the data does not permit this level of analysis–but we speculate that 2P shots are more likely to be contested than 3P shots and are therefore more likely to be subject to referee bias from the home crowd [1, 3, 9–10]. Following this idea, free throw attempts (FTA) serve as a reasonable proxy for the number of fouls called by referees in a game. Bootstrap testing suggests a statistically significant difference in home and away FTA in our dataset (home - home opp; away - away opp) at the 99% confidence level. A home team attempts 106.6 more free throws each season than away teams, which translates to roughly 1.3 more attempts per game. With the home advantage estimated at 3.24 points per game [8], this difference is non-negligible. Examining the potential influence of ref bias on home advantage would be an excellent candidate for future investigation which might be accomplished through effective use of modern video technologies.

Additional limitations to the study include: 1) The use of season attendance and populations statistics as a proxy for crowd noise and market size. As mentioned in [13], the effects of crowd noise on the advantage are complex and potentially influenced by the popularity of the opponent. If this is true, season attendance statistics may be unable to capture these effects. 2) Blocks, fouls, and steals are excluded from the analysis. Future work should examine if these variables contribute to the advantage. This is especially true for foul statistics which are directly influenced by a referee’s decision. 3) The use of season performance statistics cannot determine *when* certain shots are more important to the advantage during a game. It is reasonable to think this could be associated with the high home advantage observed in the first quarter of games and when the home team trails [10, 12]. Future analyses, particularly those examining the potential influence of referee decisions, should consider using datasets that provide in-game context.

## Supporting information

S1 Data The dataset used in the study. (JMP)

S2 Data Descriptions of the dataset used in the study. (DOCX)

S1 Fig Histogram of the observed home advantage. The distribution of the observed home advantage. The mean is 0.225 with a standard deviation of 0,1185.
(TIF)

S1 Table Era definitions. NBA eras definitions used to select training and cross validation datasets.
(TIF)
