# Supplementary material for: NBA team home advantage: Identifying key factors using an artificial neural network
Source: PLoS One. 2019 Jul 31;14(7):e0220630. doi: 10.1371/journal.pone.0220630 (PMC6668839; doi:10.1371/journal.pone.0220630)
Supplement: S2 Data — (DOCX) [file pone.0220630.s002.docx]

**Metadata**

The following is a list of metadata for the study on NBA home advantage. The basketball performance statistics were taken from basketball reference.com, attendance stats from espn.com, elevation data from USGS, and population data from census.com. (See manuscript for details). Home advantage and neural network columns were calculated here. Each column is described below:

All the following are season summary statistics for each NBA team:

NBA Era- See supplemental information (S2) for details.

Home Team FG- Field goals made by the home team in that season.

Home Team FGA- Field goals made by the home team in that season.

Home Team FG%- Percent of field goals made (attempts/makes) by the home team that season.

Home Team 2P- Two-point shots made by the home team in that season.

Home Team 2PA- Two-point shots by the home team in that season.

Home Team 2P%- Percent of two-point shots made (attempts/makes) by the home team that season.

Home Team 3P- Three-point shots made by the home team in that season.

Home Team 3PA- Three-point shots made by the home team in that season.

Home Team 3P%- Percent of three-point shots made (attempts/makes) by the home team that season.

Home Opp FG- Field goals made by the home opponent in that season.

Home Opp FGA- Field goals made by the home opponent in that season.

Home Opp FG%- Percent of field goals made (attempts/makes) by the home opponent that season.

Home Opp 2P- Two-point shots made by the home opponent in that season.

Home Opp 2PA- Two-point shots by the home opponent in that season.

Home Opp 2P%- Percent of two-point shots made (attempts/makes) by the home opponent.

Home Opp 3P- Three-point shots made by the home opponent in that season.

Home Opp 3PA- Three-point shots made by the home opponent in that season.

Home Opp 3P%- Percent of three-point shots made (attempts/makes) by the home opponent.

Away Team FG- Field goals made by the away team in that season.

Away Team FGA- Field goals made by the away team in that season.

Away Team FG%- Percent of field goals made (attempts/makes) by the away team that season.

Away Team 2P- Two-point shots made by the away team in that season.

Away Team 2PA- Two-point shots by the away team in that season.

Away Team 2P%- Percent of two-point shots made (attempts/makes) by the away team that season.

Away Team 3P- Three-point shots made by the away team in that season.

Away Team 3PA- Three-point shots made by the away team in that season.

Away Team 3P%- Percent of three-point shots made (attempts/makes) by the away team that season.

Away Opp FG- Field goals made by the away opponent in that season.

Away Opp FGA- Field goals made by the away opponent in that season.

Away Opp FG%- Percent of field goals made (attempts/makes) by the away opponent that season.

Away Opp 2P- Two-point shots made by the away opponent in that season.

Away Opp 2PA- Two-point shots by the away opponent in that season.

Away Opp 2P%- Percent of two-point shots made (attempts/makes) by the away opponent.

Away Opp 3P- Three-point shots made by the away opponent in that season.

Away Opp 3PA- Three-point shots made by the away opponent in that season.

Away Opp 3P%- Percent of three-point shots made (attempts/makes) by the away opponent.

Time Zone: 1 EST, 2 CST, 3 MST, 4, PST

For Tr/Cv: Random separation of rows into training and cross validation datasets.

Hidden layers: Used to calculate ANN.
