# Supplementary figures and images for: NBA team home advantage: Identifying key factors using an artificial neural network
Source: PLoS One. 2019 Jul 31;14(7):e0220630. doi: 10.1371/journal.pone.0220630 (PMC6668839; doi:10.1371/journal.pone.0220630)

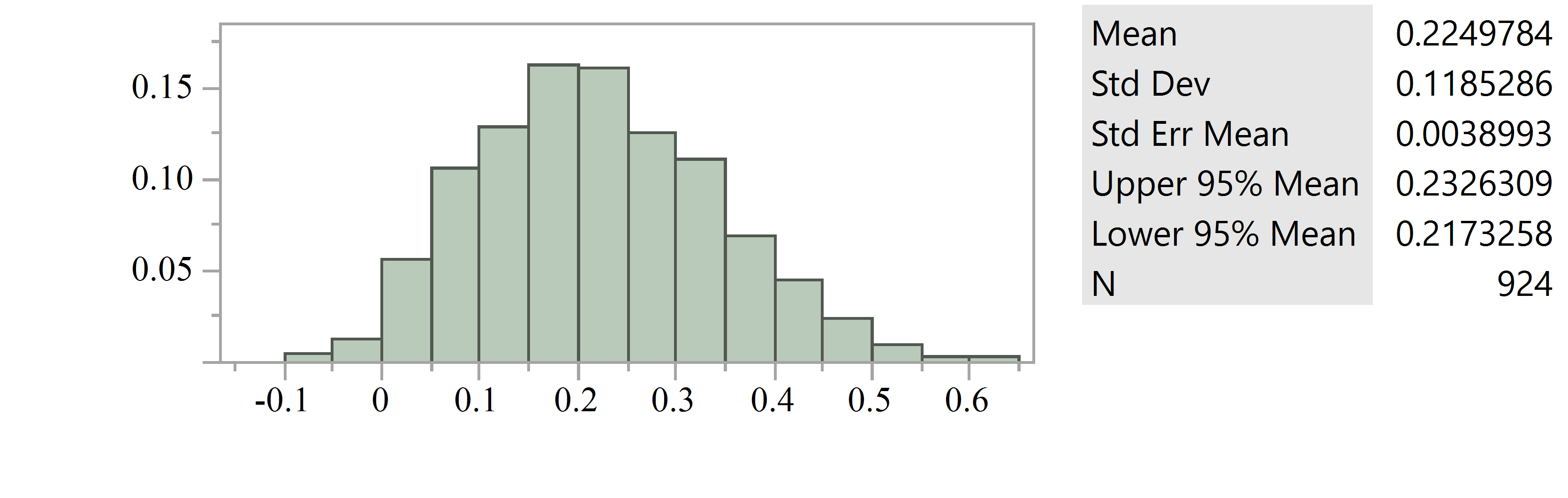

Supplement: S1 Fig — The distribution of the observed home advantage. The mean is 0.225 with a standard deviation of 0,1185. (TIF) [file pone.0220630.s003.tif]

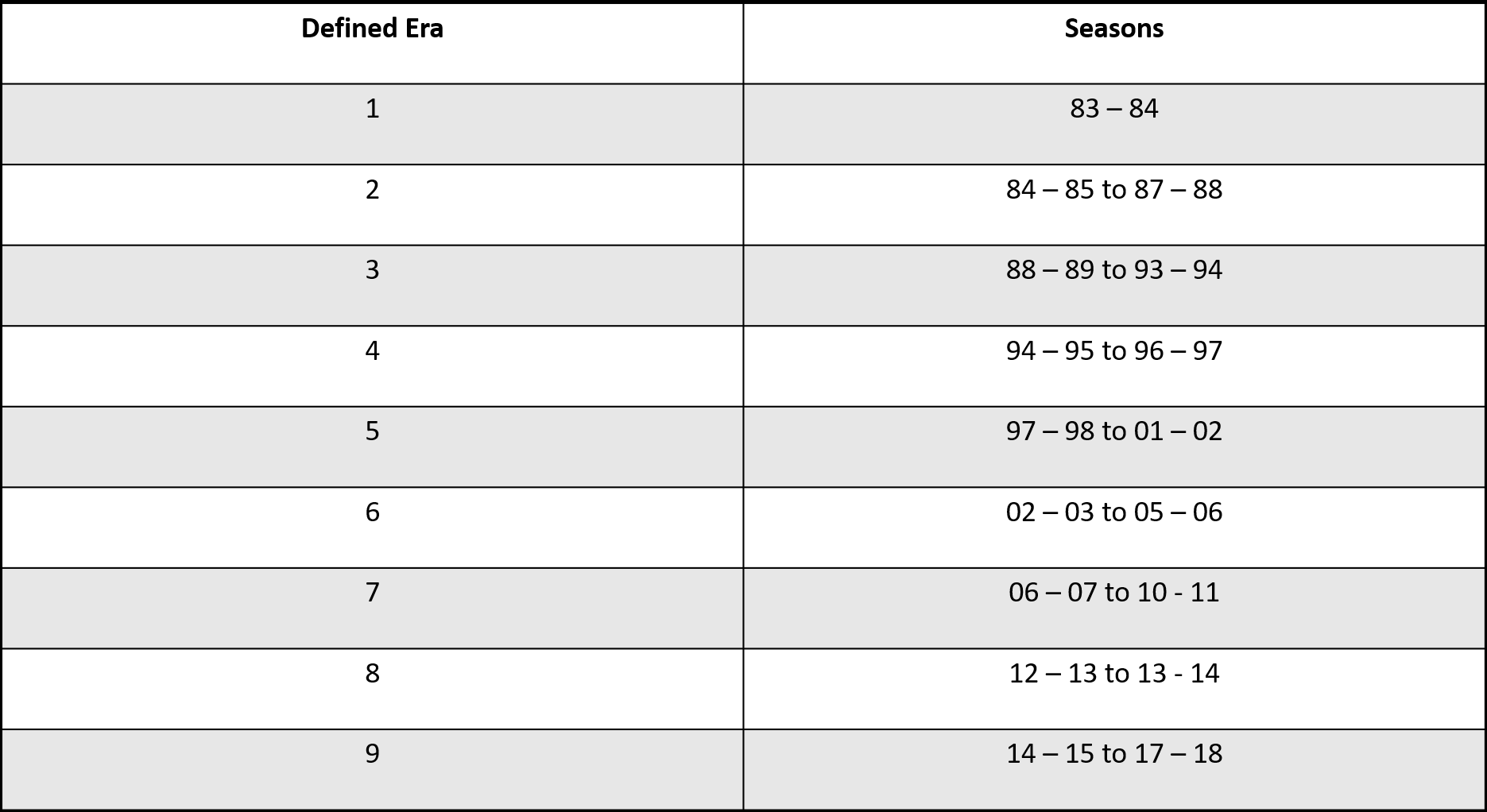

Supplement: S1 Table — NBA eras definitions used to select training and cross validation datasets. (TIF) [file pone.0220630.s004.tif]
